# Lymphangioma: Is intralesional bleomycin sclerotherapy effective?

**DOI:** 10.2349/biij.7.3.e18

**Published:** 2011-07-01

**Authors:** Z Rozman, RR Thambidorai, AM Zaleha, Z Zakaria, MA Zulfiqar

**Affiliations:** 1 Department of Radiology, Faculty of Medicine, Universiti Kebangsaan Malaysia Medical Centre, Kuala Lumpur. Malaysia; 2 Formerly, Department of Surgery, Faculty of Medicine, Universiti Kebangsaan Malaysia Medical Centre, Kuala Lumpur. Malaysia; 3 Department of Radiology, Institute of Paediatrics, Hospital Kuala Lumpur, Malaysia; 4 Department of Surgery, Institute of Paediatrics, Hospital Kuala Lumpur, Malaysia

**Keywords:** Lymphangioma, sclerotherapy, bleomycin

## Abstract

**Purpose:**

This study aims to evaluate the effectiveness of intralesional bleomycin sclerotherapy in the treatment of lymphangioma in children and to determine the incidence of complications in the treatment.

**Methods:**

This is a retrospective study of 24 children diagnosed with lymphangioma and treated with intralesional injection of bleomycin aqueous solution from January 1999 to December 2004.

**Results:**

Complete resolution was seen in 63% (15/24) of lesions, 21% (5/24) had good response and 16% (4/24) had poor response. The tumour recurred in 2 patients. Two other patients had abscess formation at the site of injection. No other serious complications or side effects were observed.

**Conclusion:**

Intralesional bleomycin therapy was very effective in the treatment of lymphangioma. Our results were comparable to other published studies. Bleomycin administered as intralesional injection was found to be safe as there was no serious complication or side effect observed in this study.

## INTRODUCTION

Lymphangioma is a benign slow growing tumour of lymphatic vessels. It may arise in any organ or soft tissue. The most frequent occurrence is in the head and neck region and involves the axilla and mediastinum. This tumour is mainly confined to the paediatric age group, but occurrences have been reported in adults. It manifests at birth in up to 65% and presents by the age of 2 years in 80–90% of cases [1]. The incidence of lymphangioma is reported to be from 1.5–2.8 per 1000, and it has no predilection for either sex or any race. The most common symptoms are swelling and cosmetic deformity. A large lesion in the neck can compress vital structures, cause respiratory obstruction, dysphagia and symptoms of nerve compression.

Intralesional sclerotherapy has become an acceptable method of treatment for lymphangiomas in children. It involves the use of a sclerosing agent that causes irritation of the endothelial lining of the lymphangioma, which leads to inflammation, fibrosis and involution [2]. Other modes of treatment such as surgical resection, incision and drainage, and radiation therapy have produced unsatisfactory results. Surgical resection for complete removal in many cases is impossible due to the nature of the lesion, which has a propensity to infiltrate tissue planes and encircle important neurovascular structures. Tumour recurrences and nerve injuries are common complications following surgery.

Previously reserved for treatment of unresectable lymphangiomas or in cases of tumour recurrence following surgery, intralesional sclerotherapy has gained popularity over recent years. Several studies using bleomycin and a newer agent OK-432 as sclerosants, have shown that this method of treatment produces favourable results compared to surgery.

We have been using bleomycin for many years, and the primary reason for choosing to use bleomycin is because it was readily available while OK432 was not yet available in Malaysia then. Our initial experience with 11 children with neck lymphangioma indicated that the drug was very effective [3]. This has led to a review of a larger series that involved childhood lymphangioma not just at the neck but also at other areas of the body.

## METHODS

This was a retrospective review of 24 successive cases of lymphangioma referred for percutaneous intralesional bleomycin sclerotherapy. The procedures were conducted in the Radiology Departments of two tertiary centres in Kuala Lumpur between January 1999 and December 2004.

The diagnosis of lymphangioma in these patients was made on the basis of clinical examination and imaging findings [4], mainly ultrasound (USG) and computed tomography (CT), and in some cases magnetic resonance imaging (MRI). Ultrasonographic assessment was performed on all patients.

**Figure 1:**
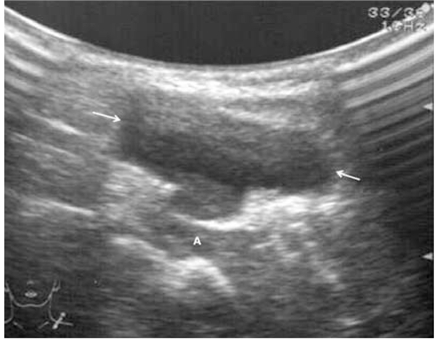
Macrocystic lymphangioma that is completely cystic (arrows).

**Figure 2:**
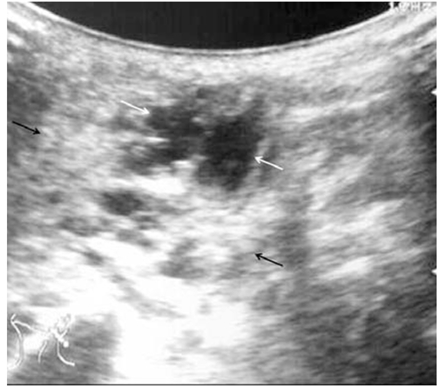
Mixed type of lymphangioma with cysts (white arrows) and echogenic areas representing microcysts (black arrows).

**Figure 3:**
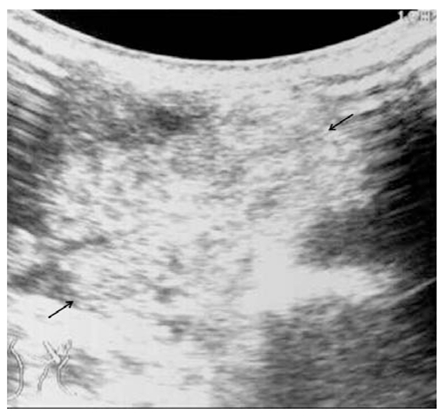
Microcystic lymphangioma that is echogenic (arrows). The echogenic appearance of the mass is due to multiple interfaces from the walls of tiny cysts.

Ultrasound was used mainly to confirm the diagnosis and to classify the type of lymphangioma. The tumours were classified based on the ultrasonographic appearance into three different subtypes: macrocystic, mixed and microcystic lymphangiomas (Figure 1–3). Patients with predominantly microcystic type of lymphangioma where the cysts were less than 1 cm in diameter were excluded from sclerotherapy. Colour Doppler was used to confirm absence of flow. As this was a review of cases performed at two centres, the ultrasound machines and transducers used were variable. Either a 5 or 7.5 MHz linear or convex probe was used.

CT or MRI was performed to ensure that the lesion was not a haemangioma, which is known to produce negative Doppler flow but will be enhanced on CT and MRI. The other role was to visualise the extent of a lesion that had involved the parapharyngeal space and mediastinum. The choice of CT or MRI depended on availability. The authors preferred CT because it is fast and could be done with sedation. MRI has the advantage of not using ionising radiation but the long procedure required general anaesthesia in the younger children. In some cases the choice of doing CT or MRI was made by the referring doctor and not by the authors.

Patients’ age, sex, ethnicity, body weight, symptoms, lesion location and lesion size were recorded. Informed consent for intralesional bleomycin therapy was obtained from the guardian.

**Figure 4:**
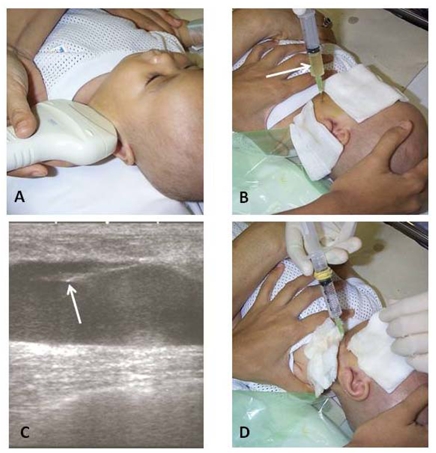
Intralesional bleomycin therapy. (A) Ultrasound assessment before injection. (B) Aspiration of cyst content reveals clear serous fluid (arrow). (C) Ultrasound image showing the tip of aspirating needle (arrow) within the cyst, and (D), while keeping the tip of the needle within the cyst, bleomycin aqueous solution is administered.

Under the effect of sedation (oral chloral hydrate with IV pethidine added when necessary) and local anaesthetic and with strict aseptic precaution, the cystic components of the tumour were aspirated with a hypodermic syringe and 23G needle using ultrasonographic guidance. While keeping the tip of aspiration needle within a cyst lumen, 0.5 mg per kg body weight of bleomycin aqueous solution (1.5 mg/ml water) was injected (Figure 4). When more than one cyst was aspirated, the calculated dose was divided by the number of cysts aspirated and the divided dose was injected into each cyst [3]. This procedure was repeated after 4 weeks if the cystic component persisted and measured at least 1 cm in diameter. The maximum cumulative dose of bleomycin allowed was 5 mg/kg body weight. The same paediatric radiologist at the two centres conducted the procedures.

Patients who failed sedation were re-scheduled for sclerotherapy under general anaesthesia (GA). Sclerotherapy for patients with stridor or respiratory distress were performed under GA.

Patients who had lesions extending into the anterior-superior mediastinum had bleomycin injection into the neck and axilla components only.

Patients were admitted for at least 24 hours following the procedure and monitored for possible immediate and delayed complications of the treatment. After discharge, patients were followed-up after 2 to 4 weeks when clinical assessment and measurements were made. If the lesion was still present, it was assessed with USG to see if further intralesional bleomycin was feasible. A repeat procedure was done when there were cysts that measured at least 1 cm in diameter. This was the minimum cyst size where the radiologists felt confident to aspirate and maintain tip of the needle within the cyst lumen for the bleomycin injection.

Follow-up and additional injections were given until the lesion completely resolved or intralesional bleomycin was no longer feasible (cysts measured less than 1 cm). The response was graded as complete resolution (total disappearance), good response (showing > 50% reduction in size) and poor response (showing < 50% reduction or no change in size). Recurrence was defined as reappearance of the tumour after complete resolution or increase in size after initial significant reduction in size. In one centre, photographs were taken at follow-up for ease of comparison. When bleomycin sclerotherapy was no longer required or no longer feasible, the follow-up period was increased to 3 monthly, then 6 monthly until the size of the lesion was stable, and then yearly [3].

Patients who showed poor response were offered surgery or sclerotherapy with OK432 when it became available in Malaysia.

## RESULTS

The 24 patients were aged between 1 day and 12 years. All patients had a swelling (n = 24). Other symptoms included stridor (n = 3), difficulty in breathing (n = 2), difficulty in swallowing (n = 1), pain (n = 2), tenderness (n = 1), and skin erythema (n = 1) (Table 1). The most common site of lesion was the neck (n = 13), followed by face (n = 5), axilla (n = 5), chest (n = 4), mediastinum (n = 4), buttock (n = 1) and thigh (n = 1) (Table 1). Lesions of the face were sited over the pre- and post-auricular regions. Chest lesions were sited at the sub-clavicular region. Mediastinal extensions were only into the anterio-superior mediastinum. The number of procedures per patient varied from once to 6 times, with a cumulative dose of 1 to 20 mg bleomycin (Table 2). Total follow-up period from the time of 1^st^ injection ranged from 2 to 42 months (Table 2).

**Table 1:** Clinical data.

**Case No.**	**Age at diagnosis, Sex, Ethnicity, body weight at diagnosis (kg)**	**Symptoms**	**Lesion location, size (cm)**	**Lesion type**
1	2 y, F, Ma, 14 kg	swelling	chest, axilla, 5 × 4 × 4	mixed
2	1 y, M, C, 8 kg	swelling	axilla, 6 × 6 × 2.5	macro
3	11 y, F, Ma, 32 kg	swelling, pain	face, 3 × 2 × 2	macro
4	1 y, F, Ma, 10 kg	swelling, pain, difficulty swallowing	neck, mediastinum, 10 × 4 × 6.5	mixed
5	8 mo, M, Ma, 9 kg	swelling	face, 3 × 3 × 2.5	mixed
6	6 mo, F, C, 8 kg	swelling	face, 5 × 4 × 4	macro
7	6 mo, M, Ma, 10 kg	swelling	neck, 4 × 2 × 2.5	mixed
8	2 y, M, C, 10 kg	swelling	chest, 6 × 4 × 4.5	macro
9	3 mo, M, Ma, 6 kg	swelling	face, 8 × 7 × 7	mixed
10	1 y 9 mo, F, I, 12 kg	swelling	neck, 3 × 3 × 2.5	macro
11	1 y 8 mo, M, Ma, 9 kg	swelling, tender	buttock, 5 × 5 × 4.5	mixed
12	5 y, F, I, 19 kg	swelling	chest, axilla, 10 × 8 × 8.5	macro
13	8 mo, F, Ma, 12 kg	swelling	neck, 3 × 2 × 2	macro
14	2 y, M, Ma, 9 kg	swelling, skin erythema	neck, face, 12 × 10 × 10.5	mixed
15	4 d, F, Ma, 7 kg	swelling	neck, 8 × 6 × 6.5	macro
16	3 y, M, Ma, 13 kg	swelling, difficulty in breathing during sleep	neck, 6 × 5 × 5	macro
17	9 mo, M, C, 9 kg	swelling, positional stridor	neck, axilla, mediastinum,15 × 15 × 14.5	mixed
18	11 d, F, Ma, 3 kg	swelling, stridor	neck, chest, mediastinum, 9 × 8 × 8	mixed
19	1 mo, F, Ma, 4 kg	swelling, stridor	neck, mediastinum, 12 × 10 × 10.5	micro
20	1 y 7 mo, F, Ma, 9 kg	swelling	neck, 6 × 6 × 6	mixed
21	1 d, F, C, 3 kg	swelling, stridor, respiratory distress	neck, 13 × 7 × 9.5	micro
22	8 y, M, C, 28 kg	swelling	thigh, 5 × 4 × 4	macro
23	12 y, M, Ma, 33 kg	swelling	neck, 8 × 8 × 8	micro
24	2 y 2 mo, F, Ma, 12 kg	swelling	axilla, 9 × 10 × 9	macro

Abbreviations y (years), mo ( months), d (days), kg (kilogram), Ma (Malay ), C (Chinese), I (Indian), macro (macrocystic), micro (microcystic)

**Table 2:** Treatment and outcome.

**Case No.**	**Age at diagnosis, Sex, Ethnicity, body weight at diagnosis (kg)**	**Lesion type**	**No. of injections**	**Total dose of bleomycin (mg)**	**Sedation (S) or GA**	**Total follow-up period from 1^st^ injection (mo)**	**Result**
1	2 y, F, Ma, 14 kg	mixed	2	17	S	2, defaulted. 2, after 2^nd^ injection	Good
2	1 y, M, C, 8 kg	macro	1	3	S	15	Resolved
3	11 y, F, Ma, 32 kg	macro	1	15	S	4	Good
4	1 y, F, Ma, 10 kg	mixed	2	15	GA	3, defaulted. 22, after 2^nd^ injection	Resolved
5	8 mo, M, Ma, 9 kg	mixed	2	6	S	3	Resolved
6	6 mo, F, C, 8 kg	macro	1	5	S	7	Resolved
7	6 mo, M, Ma, 10 kg	mixed	1	4	S	17	Resolved
8	2 y, M, C, 10 kg	macro	1	5	S	7	Resolved
9	3 mo, M, Ma, 6 kg	mixed	3	8	S	12	Good
10	1 y 9 mo, F, I, 12 kg	macro	3	17	S	7	Resolved
11	1 y 8 mo, M, Ma, 9 kg	mixed	1	5	S	11	Resolved
12	5 y, F, I, 19 kg	macro	2	20	S, GA	11	Resolved
13	8 mo, F, Ma, 12 kg	macro	2	6	S	4	Resolved
14	2 y, M, Ma, 9 kg	mixed	2	6	S	42	Resolved
15	4 d, F, Ma, 7 kg	macro	1	3	S	2	Resolved
16	3 y, M, Ma, 13 kg	macro	1	5	S	2	Resolved
17	9 mo, M, C, 9 kg	mixed	6	18	S	8	Poor
18	11 d, F, Ma, 3 kg	mixed	3	5	GA	16	Good
19	1 mo, F, Ma, 4 kg	micro	1	1	GA	7	Poor
20	1 y 7 mo, F, Ma, 9 kg	mixed	2	9	S	7	Good
21	1 d, F, C, 3 kg	micro	5	8	GA	4	Poor
22	8 y, M, C, 28 kg	macro	1	14	S	30	Resolved
23	12 y, M, Ma, 33 kg	micro	1	17	S	5	Poor
24	2 y 2 mo, F, Ma, 12 kg	macro	1	4	S	5	Resolved

Abbreviations: y (years), mo ( months), d (days), kg (kilogram), Ma (Malay ), C (Chinese), I (Indian), macro (macrocystic), micro (microcystic)

**Figure 5:**
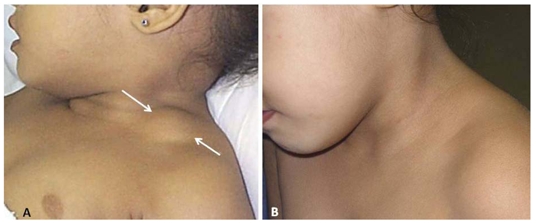
(A) A 2-year-old girl (Case 10) with left supraclavicular lymphangioma (arrows) which had been present for 1 year. This patient’s ultrasound is shown in Figure 1. (B) Complete regression. An excellent result is achieved 1 year later, after 3 injections of bleomycin.

**Figure 6:**
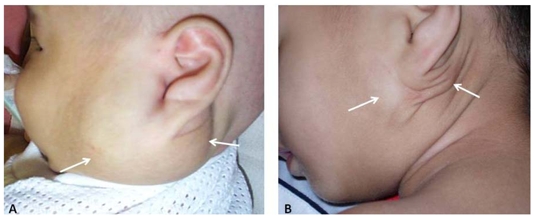
(A) A large disfiguring lymphangioma over the angle of left mandible (arrows) in a 3-month-old baby (Case 9). This patient’s ultrasound image is shown in Figure 2. (B) One year later, the appearance after three injections of bleomycin, significant reduction in size was observed. A small swelling (arrows) was noticeable in the pre- and post-auricular area.

**Figure 7:**
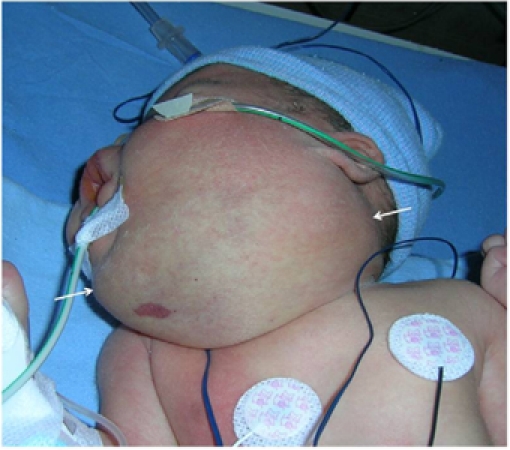
Poor response. This patient’s ultrasound is shown in Figure 3. This baby with a massive neck mass had respiratory distress at birth (Case 21). She was intubated and monitored in the intensive care unit. Imaging studies confirmed the swelling as predominantly microcystic type of lymphangioma. The lesion failed to regress after 6 injections of bleomycin.

Complete resolution (Figure 5) was seen in 63% (15/24) of lesions, 20% (5/24) had good response (Figure 6) and the remaining 17% (4/24) had poor response (Figure 7). The observations were recorded at the end of follow-up or at the time the patient was last seen in the clinic, as documented in the case notes (Table 2).

Intralesional bleomycin therapy failed in 4 patients (Table 2). The first patient who failed sclerotherapy (Case 17) had six injections and minimal reduction in size was achieved. Further, sclerotherapy could not be performed because this patient had mixed type of lymphangioma. After six injections, there were no further cysts large enough for aspiration and injection. This patient subsequently underwent surgical resection and 80% of the tumour mass was removed. The second patient who responded poorly (Case 19) had a predominantly microcystic type of lymphangioma and only one cyst was aspirated and injected. Subsequently, this patient underwent three intralesional OK-432 injections. This drug therapy had also failed to reduce the size of the lesion and the patient underwent surgical resection. The third patient was a newborn who presented with a huge neck mass and had respiratory distress during postnatal period (Case 21). She was intubated and monitored in the Intensive Care Unit. After five injections of bleomycin, there was only a slight reduction in the size of the mass that was predominantly microcystic. However, the patient became well enough to be extubated and went home. The fourth subject had a swelling in the right neck at birth (Case 23). Surgical excision was performed at the age of 1 month. The patient presented later, at the age of 12 years with a recurrent swelling measuring 8 × 8 cm. Intralesional bleomycin was given only once, as in the subsequent follow-up, the remaining lesion was the microcystic component and was not suitable for further bleomycin treatment. Further surgical excision was contemplated but the patient defaulted follow-up.

There were 2 (8%) recurrences. Both patients (Cases 1 and 4) had good response (> 50% reduction) after one injection of bleomycin. Both defaulted follow-up and presented 1 year later with a swelling that was bigger than initial post-injection size. Case 1 had good response after another injection of bleomycin. The lesion in Case 4 completely resolved after another injection of bleomycin.

Comparing the response rate with the lesion type, 10 out of 11 macrocystic lesions had complete resolution and only one had a good partial response. Complete resolution was seen in 5 of 10 mixed lesions. The other 4 mixed lesions had good partial response and the remaining 1 had poor response. All 3 predominantly microcystic type responded poorly to the treatment (Table 3).

**Table 3:** Outcome of Bleomycin Sclerotherapy.

**Outcome**	**Type of Lymphangioma**			
	**Macrocysticn (%)**	**Mixedn (%)**	**Microcysticn (%)**	**Totaln (%)**
Complete resolution	10 (42%)	5 (21%)	0	15 (63%)
Good response (>50% reduction in size)	1 (4%)	4 (16%)	0	5 (20%)
Poor response (<50% reduction in size)	0	1 (4%)	3 (13%)	4 (17%)
Total	11 (46%)	10 (41%)	3 (13%)	24 (100%)

Immediate complications reported include skin erythema at injection site, local swelling, mild tenderness and fever, which were controlled by oral antipyretics. One patient had excessive vomiting from the effect of IV sedation. However, no life- threatening complications such as respiratory obstruction and severe hypersensitivity reaction to bleomycin were observed. Two patients, Cases 7 and 18 (Table 2), developed an abscess at the site of injection, 7 months and 1 month respectively after the last injection. Fistula formation and signs related to peripheral nerve damage were not observed. No patient developed excessive scarring as a result of the procedure.

## DISCUSSION

The idea of using sclerotherapy in the treatment of lymphangioma occurred when it was noted that lymphatic malformations spontaneously involute when they became infected and the infection resolved. The first case of lymphangioma treated by sclerotherapy was reported in 1933, using sodium morrhuate. Complete tumour regression was noted in 6 weeks following intralesional injection [1]. Since then various sclerosing agents have been used, namely iodine, ethanolamine oleate, alcohol, ethibloc, tetracycline and cyclophosphamide. Bleomycin and OK-432 are currently the most popular sclerosants. However, no studies have been conducted to compare the efficacy between these two agents.

Bleomycin is an anti-neoplastic antibiotic, produced by the fermentation of streptomyces verticillus. Discovered in 1965, this drug was found to cause single- and double-strand DNA breaks and inhibition of DNA and RNA synthesis [1]. Since then it has been used for its anti-neoplastic property to treat malignancy. In the treatment of malignant pleural effusion, it was observed that bleomycin caused marked fibrosis and scarring. This sclerosing property was first put into use in the treatment of lymphatic malformation in 1977. In this series, 8 patients who either had incomplete surgical resection, recurrent or non-resectable tumour were treated with bleomycin. Good results were reported in these patients [1].

Studies using bleomycin have produced promising results [1, 3, 5–7]. Our study indicated that intralesional bleomycin therapy was very effective and the results were comparable to these published series. Different authors have quoted success rates of between 36 to 63% for complete tumour regression, of up to 88% significant lesion regression, and poor response of between 12 to 23% using either bleomycin or OK432 (Table 4).

**Table 4:** Results of Series using Bleomycin and OK432.

**Study**	**Year**	**No. of patients**	**Significant regression (>50-100% resolution)**	**Complete Resolution (100% resolution)**	**Poor/No Response (<50% resolution)**	**Recurrence**
**Bleomycin**						
Rozman		24	84% (20)	63% (15)	16% (4)	(2)
Mahajan [7]	2004	15	86.7% (13)	53.3% (8)	13.3% (2)	-
Zulfiqar [3]	1999	11	82% (9)	36% (4)	18% (2)	-
Orford [6]	1995	16	88% (14)	44% (7)	12% (2)	(1)
Okada [1]	1992	29	86% (25)	55% (16)	14% (4)	(3)
Tanigawa [5]	1987	33	82% (27)	39.4% (13)	18% (6)	-
**OK-432**						
Ruiz Jr. [10]	2004	19	84%	63%	16%	-
Nielsen [9]	2003	13	77%	46%	23%	-
Ogita [8]	1991	23	78%	43%	22%	-

In line with other published data, we found that macrocystic and mixed type of lymphangiomas were the ones that responded well to the treatment. In the mixed and predominantly microcystic type, once the bigger cysts have been aspirated, leaving only small cysts and microscopic lymphatic channels, further aspiration could not be performed. This was seen in four of the mixed lesions. However, the lesions reduced to a size that was cosmetically acceptable. All our predominantly microcystic lesions and one mixed lesion resulted in only slight reduction in size following sclerotherapy. This small size reduction however, would facilitate surgery if indicated.

In this study, bleomycin aqueous solution was injected directly into a cyst after aspiration of the cyst content. Unlike OK-432, most authors advise against injecting bleomycin into the microcystic part or intraprenchymal component of the tumour. Intraparenchymal injection has higher rate of systemic absorption, hence, greater risk of systemic toxicity. This is where OK-432 has an advantage over bleomycin. Intraparenchymal injection of OK-432 was performed without significant clinical side effects. Thus, this drug can be used to treat microcystic lymphangioma [8–10].

Two recurrences of tumour were observed in this study. We defined recurrence as reappearance of the tumour after complete resolution or increase in size after initial significant reduction in size was seen. Both these patients had defaulted post-injection assessments. One further injection was given and one patient had complete resolution while the other had good response. The true rate of recurrences, however, could only be ascertained if patients were followed-up for a longer period of time.

There were four patients with lesions that extended into the anterior-superior mediastinum where only the neck and axilla components were aspirated and injected with bleomycin. With this approach it was hoped that as the neck component reduced in size, the mediastinal component would retract into the neck. The mediastinal component were not directly aspirated and injected for fear that it could cause mediastinitis and compromise adjacent vital structures. In the 1^st^ reported case of mediastinal lymphangioma treated with percutaneous sclerotherapy, Ethibloc and absolute ethanol were used as sclerosants. In this case, the giant lymphangioma was located in the retrocardiac region [11].

No serious complications were seen as a result of intralesional bleomycin therapy. Most patients complained of mild skin erythema, local swelling, induration, mild tenderness and fever. These symptoms lasted for a day or two but did not prolong their stay in hospital. In patients with large neck lesions and airway compromise was expected, support from the intensive care team was summoned. In these patients, intralesional injection was performed under general anaesthesia with their airway secured. The patients remained intubated post-injection and monitored in the ICU for 24 hours.

In patients with complete resolution, there was no undesired excessive scarring or pigmentation noted at the injection site. We believe that the aesthetic result from the use of bleomycin was as good as what was claimed from the use of OK-432.

Local infections at the site of injections were seen in 2 out of 24 patients. The abscess occurred 7 months and at 1 month after the last bleomycin injection. Incision and drainage were performed and later the lymphangioma resolved completely in 1 patient. Whether these infections occurred as a result of the sclerotherapy or were de-novo abscesses from the lymphangioma, remains unanswered. A significant time gap was observed between the last injection and the abscess formation. We, therefore, do not think that this complication was related to our procedure.

The primary concern of bleomycin therapy is its risk of pulmonary toxicity. The risk is dose related with an increased incidence associated with a total dose exceeding 400 i.u. or a single dose exceeding 30 mg/m^2^ of body surface area given intra-venously to oncology patients [12]. Elderly oncology patients and those with underlying pulmonary disease and renal failure were at greater risk. However, the doses used in sclerotherapy were much lower than those used for oncology purposes. Clinical studies from Japan, India, Australia and Malaysia in which a total of 104 patients were treated with intralesional bleomycin did not report pulmonary fibrosis as a complication [1, 3, 5–7].

There was no report comparing the efficacy of the various sclerosing agents in a single study. Several published series were all review studies using a single agent. Double-blind, case-control clinical trials comparing these sclerosing agents in the treatment of lymphangioma would be difficult. This is due to the following: limited cases as there is low incidence of the tumour, complexity of the tumour, long follow-up after treatment, and the high rate of confounders that would contribute to biases of the obtained results. Lesion subtypes, its location, size and presence of complication could all affect the outcomes. All these confounders need to be controlled if statistically significant results were to be obtained. This would mean a very large number of cases need to be recruited and would involve many different centres. A single series of sclerotherapy treatment of lymphangioma usually would not achieve statistically significant results. Looking at data of these small series together, however, may produce more concrete results. Solid conclusions must therefore be drawn from reviewing many different series as a whole.

## CONCLUSION

Intralesional bleomycin therapy is a safe and effective method of treatment for lymphangioma. The results from our series are comparable with other published series. No serious side effects from intralesional bleomycin therapy were observed. Our results, together with the results from other published series support the continued use of sclerotherapy with bleomycin in the treatment of lymphangioma. Sclerotherapy is recommended in place of surgery as the first-line treatment modality. The latter must be reserved for those lesions where sclerotherapy had failed. Which sclerotherapy agent is superior? This question may only be answered by reviewing the results of various case series using the different sclerosing agents.
